# Hyperdynamic left ventricular ejection fraction in the intensive care unit

**DOI:** 10.1186/s13054-015-1012-8

**Published:** 2015-08-07

**Authors:** Joseph R. Paonessa, Thomas Brennan, Marco Pimentel, Daniel Steinhaus, Mengling Feng, Leo Anthony Celi

**Affiliations:** Beth Israel Deaconess Medical Center, 330 Brookline Avenue, Boston, MA 02215 USA; Harvard Medical School, 25 Shattuck Street, Boston, MA 02115 USA; Massachusetts Institute of Technology, 32 Vassar Street, Cambridge, MA 02139 USA; Institute of Biomedical Engineering, Oxford University, Headington, Oxford, OX3 7DQ UK; Present address: 1122 N. Clark Street, Apt. 3709, Chicago, IL 60610 USA

## Abstract

**Introduction:**

Limited information exists on the etiology, prevalence, and significance of hyperdynamic left ventricular ejection fraction (HDLVEF) in the intensive care unit (ICU). Our aim in the present study was to compare characteristics and outcomes of patients with HDLVEF with those of patients with normal left ventricular ejection fraction in the ICU using a large, public, deidentified critical care database.

**Methods:**

We conducted a longitudinal, single-center, retrospective cohort study of adult patients who underwent echocardiography during a medical or surgical ICU admission at the Beth Israel Deaconess Medical Center using the Multiparameter Intelligent Monitoring in Intensive Care II database. The final cohort had 2867 patients, of whom 324 had HDLVEF, defined as an ejection fraction >70 %. Patients with an ejection fraction <55 % were excluded.

**Results:**

Compared with critically ill patients with normal left ventricular ejection fraction, the finding of HDLVEF in critically ill patients was associated with female sex, increased age, and the diagnoses of hypertension and cancer. Patients with HDLVEF had increased 28-day mortality compared with those with normal ejection fraction in multivariate logistic regression analysis adjusted for age, sex, Sequential Organ Failure Assessment score, Elixhauser score for comorbidities, vasopressor use, and mechanical ventilation use (odds ratio 1.38, 95 % confidence interval 1.039–1.842, *p* =0.02).

**Conclusions:**

The presence of HDLVEF portended increased 28-day mortality, and may be helpful as a gravity marker for prognosis in patients admitted to the ICU. Further research is warranted to gain a better understanding of how these patients respond to common interventions in the ICU and to determine if pharmacologic modulation of HDLVEF improves outcomes.

## Introduction

Hyperdynamic left ventricular ejection fraction (HDLVEF) on transthoracic echocardiography (TTE) is a frequent finding in the intensive care unit (ICU). The American College of Cardiology (ACC) defines HDLVEF as a left ventricular ejection fraction >70 % [1]. In spite of the growing use of TTE in the critical care setting, limited information exists on the etiology, prevalence, and significance of HDLVEF in the ICU.

Prior studies have suggested increased prevalence of HDLVEF in certain patient populations and disease processes. Patients with sepsis commonly have low systemic vascular resistance [2] and increased circulating catecholamines, leading to increased contractility [3], which could explain why patients with sepsis may develop HDLVEF. One study demonstrated that, in patients with septic shock, HDLVEF was more common in the subset of patients with concurrent cirrhosis [4]. Research has also suggested that HDLVEF in patients with non-traumatic shock is highly specific, but not sensitive, for sepsis [5]. Outside the diagnosis of sepsis, critically ill patients with HDLVEF have not been studied.

In the outpatient setting, female sex and obesity have been associated with a higher prevalence of HDLVEF without any clear mechanism [6, 7]. Furthermore, HDLVEF has not been shown to be associated with aerobic fitness [8], which suggests that HDLVEF may be due to a pathophysiologic response rather than to cardiovascular conditioning. These studies have provided some insight, but the significance of HDLVEF remains undefined.

There have not been any studies in which researchers compared outcomes of patients with HDLVEF with those of patients with normal left ventricular ejection fraction on an electrocardiogram (NLVEF). Using a large, public, deidentified critical care database, we studied the prevalence, characteristics, and outcomes of patients with HDLVEF in an ICU setting.

## Materials and methods

We conducted a longitudinal, single-center, retrospective cohort study of adult patients who underwent TTE during an ICU admission at the Beth Israel Deaconess Medical Center. Data were extracted from the Multiparameter Intelligent Monitoring in Intensive Care II database (MIMIC II). MIMIC II is freely available in the public domain and contains information derived from the electronic medical records of 32,425 patients admitted to the ICUs at the Beth Israel Deaconess Medical Center between 2001 and 2008. Twenty-eight–day mortality information was obtained from Social Security Death Index records. The creation and use of the MIMIC II database for research was approved by the institutional review boards of both Beth Israel Deaconess Medical Center and the Massachusetts Institute of Technology (Institutional Review Board protocol 2001-P-001699/3). No further patient consent is required for use of this deidentified public database.

All adult patients in the database were screened. Data regarding age, sex, Sequential Organ Failure Assessment score (SOFA) [9], laboratory values, vital signs, diagnosis-related group (DRG) codes, and International Classification of Diseases–Ninth Revision (ICD-9) diagnoses were extracted. Medical comorbidities were represented by the Elixhauser score [10] for 30 comorbidities as calculated using the DRG and ICD-9 codes from the respective hospital admission. The worst values of common pertinent laboratory results were also extracted, including white blood cell count, lactate, and creatinine. Patients with sepsis were identified using the Angus criteria [11].

For patients with multiple ICU stays, the first ICU admission was used. Patients in the database were admitted to one of the following: medical ICU (MICU), surgical ICU (SICU), cardiac ICU, or cardiac surgical resuscitation unit. The study was limited to MICU and SICU patients to exclude elective admissions. Patients with at least one TTE were included in the cohort. HDLVEF was defined as an ejection fraction (EF) >70 % based on the ACC guidelines, and NLVEF was defined as an EF of 55–70 % [1]. Those with an EF <55 % were excluded from the analysis to minimize confounding of the relationship between HDLVEF and clinical outcomes. EF was determined predominately by visual estimation using two-dimensional imaging with incorporation of fractional shortening in the parasternal long-axis view according to guidelines established by the American Society of Echocardiography [12]. Contrast echocardiography was used when standard imaging was not diagnostic, and this method has been shown to reliably correlate with quantitative measurements [13]. To ensure the quality of using natural language processing for EF categorization, a random sample of 100 TTE reports was manually reviewed. This showed exceptionally high algorithm accuracy.

Baseline comparisons were performed using Fisher’s exact test for categorical variables, where counts and percentages were reported. Continuous variables were compared using a two-sample Wilcoxon rank-sum test, also known as the Mann–Whitney *U* test, and reported as median with interquartile range (IQR). Statistical significance was defined as *p* values <0.01 for the baseline characteristics shown in Table 1.

**Table 1:** Characteristics of patients with NLVEF compared with HDLVEF

	NLVEF	HDLVEF	*p* Value
Age, yr	65 [51–78]	69 [56–78]	0.03
Male sex	1246 (49 %)	134 (41 %)	**<0.01**
MICU	1720 (68 %)	221 (68 %)	0.9
SICU	823 (32 %)	103 (32 %)	0.9
Time to echo (days)	1.1 [0.1–3.3]	0.9 [0.0–4.2]	0.4
Time to vasopressor use (days)	0.1 [0.0–0.5]	0.1 [0.0–0.7]	1.0
Comorbidities			
Diabetes mellitus	590 (23 %)	89 (27 %)	0.1
Alcohol abuse	153 (6 %)	19 (6 %)	1.0
Arrhythmias	700 (28 %)	82 (25 %)	0.4
Valvular disease	255 (10 %)	38 (12 %)	0.3
Hypertension	852 (34 %)	134 (41 %)	**<0.01**
Renal failure	213 (8 %)	29 (9 %)	0.7
Chronic pulmonary	536 (21 %)	68 (21 %)	1.0
Liver disease	199 (8 %)	32 (10 %)	0.2
Cancer	120 (5 %)	28 (9 %)	**<0.01**
Psychosis	117 (5 %)	15 (5 %)	1.0
Depression	148 (6 %)	12 (4 %)	0.1
CHF	841 (33 %)	127 (39 %)	0.03

*CHF* congestive heart failure, *HDLVEF* hyperdynamic left ventricular ejection fraction on echocardiogram, *MICU* medical intensive care unit, *NLVEF* normal left ventricular ejection fraction on echocardiogram, *SICU* surgical intensive care unit

Characteristics of patients with NLVEF compared with HDLVEF are displayed as either *n* (%) or median [IQR]. *p* Values <0.01 are listed in bold

A multivariable logistic regression analysis was performed with 28-day mortality as the outcome. Covariates included age, sex, SOFA score, Elixhauser score of comorbidities, mechanical ventilation use, vasopressor use, and the presence of HDLVEF. Mechanical ventilation was represented as a binary variable. Since vasopressors have wide ranges of therapeutic intensity, sensitivity analyses were performed in the way vasopressor use was adjusted for. The analyses were adjusted for vasopressor use as a binary variable (yes or no), maximum number of vasopressors, or maximum vasopressor dose, defined as a fraction of the highest recommended dose for each vasopressor. The odds ratios (ORs) are preserved in the logistic regression model with 95 % confidence intervals (CIs). Two-sided *p* values <0.05 were considered statistically significant.

## Results

### Inclusion and exclusion criteria

**Fig. 1:**
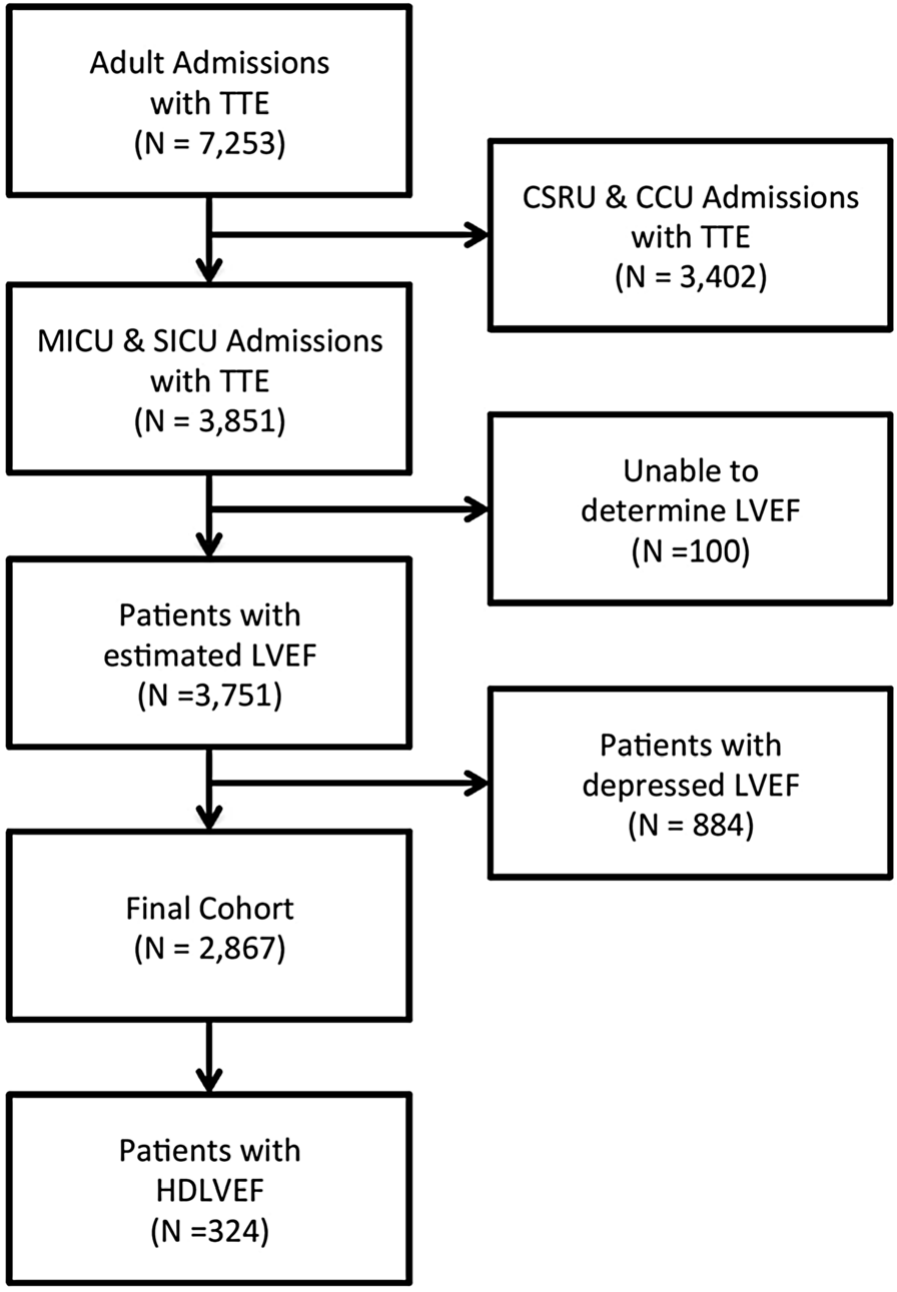
Flow diagram showing initial selection of cohort and excluded patients. *CSRU* cardiac surgical resuscitation unit, *HDLVEF* hyperdynamic left ventricular ejection fraction on echocardiogram, *LVEF* left ventricular ejection fraction, *MICU* medical intensive care unit, *SICU* surgical intensive care unit, *TTE* transthoracic echocardiography

As shown in Fig. 1, there were a total of 7253 patients with TTE. The study was limited to MICU and SICU patients to exclude elective admissions. The remaining cohort comprised 3851 patients with TTE. Per TTE reports, EF could not be estimated in 100 patients, owing to poor windows, body habitus or patient positioning. These patients were likewise excluded. Ultimately, 884 patients with depressed EF were removed. The final cohort comprised 2867 patients, of whom 324 had HDLVEF. The time to TTE was similar between the HDLVEF and NLVEF groups, with the median being approximately 1 day after arrival to ICU (Table 1).

### Study outcome analysis

The distribution of MICU and SICU patients were similar in the groups with NLVEF and HDLVEF (Table 1). With respect to baseline characteristics, patients with HDLVEF were more likely to be older and female. Diagnoses associated with HDLVEF included hypertension and cancer, based on ICD-9 codes. There was a trend toward association with congestive heart failure (CHF) and sepsis, but it did not reach a *p* value <0.01. HDLVEF patients as a cohort also had a slightly higher SOFA score (7 [IQR 4–10] vs. 6 [IQR 3–9], *p* <0.01), suggesting increased acute disease severity. Tachycardia, lower mean arterial pressure, leukocytosis, and higher serum lactates were all more commonly seen in the HDLVEF cohort. HDLVEF patients were treated more frequently with vasopressors and mechanical ventilation, as shown in Table 2. Norepinephrine, phenylephrine, and dopamine were the most frequently used vasopressors in both groups, respectively. Dobutamine and epinephrine were rarely used in both groups, and levosimendan was not used in any of the patients (data not shown). Use of inotropes is much more common in the cardiac and cardiothoracic ICU patients, who were not included in this study.

In multivariate analysis, HDLVEF patients had increased 28-day mortality compared with patients with NLVEF (OR 1.38, 95 % CI 1.039–1.842, *p* =0.02) after adjusting for age, sex, SOFA score, Elixhauser score, mechanical ventilation, and vasopressor use (Table 3). All three methods of representation of vasopressor use in the sensitivity analysis did not alter the OR of the presence of HDLVEF in the multivariable logistic regression model (data not shown).

**Table 2:** Acute illness severity and treatments performed of patients with NLVEF compared with HDLVEF

	NLVEF	HDLVEF	*p* value
Acute illness indicators			
SOFA score	6 [3–9]	7 [4–10]	**<0.01**
Sepsis	1025 (40 %)	150 (46 %)	0.04
Heart rate maximum, beats/min	113 [98–130]	120 [103–139]	**<0.01**
MAP minimum, mmHg	56 [48–64]	52 [43–62]	**<0.01**
Temperature maximum (°C)	37.8 [37.3–38.5]	37.9 [37.4–38.5]	0.6
Selected laboratory results			
WBC max k/μL	13.3 [9.4–18.3]	14.6 [10.2–20.5]	**<0.01**
Lactate max mMol/L	2.1 [1.4–3.7]	2.6 [1.6–4.8]	**<0.01**
Creat max mg/dL	1.1 [0.8–1.8]	1.2 [0.8–2.0]	0.04
Treatments received			
Renal replacement	288 (11 %)	50 (15 %)	0.04
Vasopressor use	741 (29 %)	125 (39 %)	**<0.01**
Mechanical ventilation	1298 (51 %)	198 (61 %)	**<0.01**
IV fluids, L (first 24 h)	2.2 [0.6–5.2]	2.5 [0.7–5.7]	0.2

*HDLVEF* hyperdynamic left ventricular ejection fraction on echocardiogram, *IV* intravenous, *MAP* mean arterial pressure, *NLVEF* normal left ventricular ejection fraction on echocardiogram, *SOFA* Sequential Organ Failure Assessment, *WBC* white blood cell count

Acute illness severity and treatments performed for patients with NLVEF compared with HDLVEF are displayed as either *n* (%) or median [IQR]. *p* Values <0.01 are listed in bold

**Table 3:** Multivariable logistic regression model for 28-day mortality

	Odds ratio (95 % confidence interval)	*p* value
Age	1.011 (1.007–1.016)	**<0.001**
Male sex	0.972 (0.792–1.192)	0.8
Elixhauser score	1.054 (1.037–1.071)	**<0.001**
SOFA score	1.128 (1.091–1.166)	**<0.001**
Mechanical ventilation	1.177 (0.906–1.530)	0.2
Vasopressor use	1.210 (0.943–1.549)	0.1
HDLVEF	1.389 (1.039–1.842)	**0.02**

*HDLVEF* hyperdynamic left ventricular ejection fraction on echocardiogram, *SOFA* Sequential Organ Failure Assessment

Multivariable logistic regression model for 28-day mortality in all patients. *p* Values <0.05 are listed in bold

## Discussion

HDLVEF was present in 8.6 % of ICU patients who had a TTE during their ICU admission. The median time to TTE after arrival to the ICU was similar between groups (Table 1), which suggests that the presence of HDLVEF and its association with the outcome were not confounded by the timing of TTE.

The finding of HDLVEF was associated with female sex, increased age, and diagnoses of hypertension and cancer. There was a trend toward association with CHF and sepsis. In multivariate logistic regression analysis, patients with HDLVEF had increased 28-day mortality compared with those with NLVEF.

The exact cause of HDLVEF in the ICU is not well understood. Cardiac function is extremely variable in the setting of critical illness and depends on multiple physiologic determinants [3]. Cardiac systolic function is related to heart rate, preload, afterload, and contractility. A patient’s EF may be hyperdynamic in the setting of critical illness owing to changes in these basic physiologic parameters.

Prior research suggested that hypovolemia could lead to HDLVEF [14]. In contrast, a small study showed that three of four patients with HDLVEF on transesophageal echocardiography had pulmonary capillary wedge pressures >20 mmHg, which argues against hypovolemia as an etiology [15]. In our study, patients with NLVEF and HDLVEF received a similar amount of intravenous fluid in the first 24 h (Table 1), although this cannot exclude hypovolemia and decreased systemic vascular resistance as contributors to HDLVEF.

HDLVEF was also found to be associated with cancer. Many studies have shown increased cytokine levels in patients with various types of cancer [16–19], which may explain the presence of HDLVEF among critically ill patients with cancer.

It is unclear if diastolic dysfunction contributes to the development of HDLVEF. It is plausible that patients with diastolic dysfunction are more likely to develop HDLVEF, either chronically or in the setting of acute illness, to compensate for the reduced preload from impaired relaxation during ventricular filling. Another mechanism could be increased circulating cytokines within the tumor necrosis factor α axis, which has been suggested to be a contributor to the development of CHF with preserved EF [20]. Several other biomarkers of myocyte stress, inflammation, and extracellular matrix remodeling have been shown to be associated with CHF with preserved EF [21]. It is unknown if this may be seen in HDLVEF as well. Alternatively, mismatch of myocardial contractility and arterial compliance may be present in patients with HDLVEF, which might explain the trend toward development of CHF. The presence of ventriculoarterial decoupling, defined as the ratio of the arterial elastance to the ventricular elastance, can be found in many disease states and may lead to worse outcomes [22]. These inferences cannot be deduced from our analysis and should be investigated further.

As anticipated based on a literature review, we found that HDLVEF is more frequently seen with a diagnosis of sepsis. Adequately resuscitated patients with severe sepsis can have warm peripheries, high cardiac output, and HDLVEF [23]. Despite a prior study suggesting that HDLVEF was highly specific for sepsis, our study showed that HDLVEF was seen frequently outside the diagnosis of sepsis. It is also important to note that EF may be depressed in sepsis, as has been demonstrated in prior studies [19, 24–28]. The information in the literature is mixed with regard to EF and prognosis in patients with sepsis [29, 30]. In addition to systolic dysfunction, diastolic dysfunction has been described in sepsis [26, 27]. It might contribute to the development of HDLVEF, though this present study was not designed for such an investigation.

### Limitations

Our study is limited by its retrospective nature and its inclusion of a relatively heterogeneous group of patients whose data were extracted from electronic medical records in a large clinical database. Other variables not captured in the database may account for residual confounding. Disease associations such as hypertension and cancer are based on ICD-9 codes, which can lead to inconsistent levels of reporting, but this may be somewhat mitigated by the single-center scope of the study. The indications for TTE could be variable and could not be accounted for in this study. Common reasons for TTE in our institution include workup for hypotension and heart failure and evaluation for bacterial endocarditis or right heart strain owing to acute pulmonary embolism. In this study, the need for TTE was at the discretion of the ordering physicians. The indication was not documented.

Bias might have been introduced by excluding patients without TTE during their ICU admission. We compared patients who had TTE to those without TTE with respect to the parameters listed in Table 1, in addition to mortality data. On the basis of this additional analysis, we found that the patients who did not undergo TTE were younger, had fewer comorbidities, were less sick (based on SOFA scores, vital signs, laboratory results, use of vasopressors, and mechanical ventilation), and had better outcomes (data not shown) than other patients. This makes sense from a clinical standpoint—the physicians did not feel that additional information provided by TTE was necessary to manage these patients. These patients who were excluded because they did not undergo TTE look more similar to those patients with NLVEF than to those with HDLVEF. If most of the excluded patients indeed had NLVEF, their inclusion into the analysis, assuming that they had NLVEF, would likely have increased the effect size, given their better outcomes.

### Implications

To our knowledge, this study is the first large-scale evaluation of HDLVEF in the ICU setting. It is difficult to determine exactly why HDLVEF has a worse prognosis when seen during critical illness. The etiology of increased mortality in the HDLVEF group may be related to the myocardial mechanics of HDLVEF, or it could be a marker or proxy of a pathophysiologic process. The presence of HDLVEF, in addition to traditional mortality risk predictors, may provide additional prognostic implications for ICU patients.

### Further research

HDLVEF patients may respond differently to common interventions such as fluid administration, vasopressor use, and mechanical ventilation. If cardiovascular mechanics are the etiology of worse prognosis, then further studies might be considered to investigate whether modulating HDLVEF with pharmacotherapy improves outcomes. Interestingly, β-blockade has been shown to decrease mortality in patients with severe septic shock [31]. If catecholamine surge is the etiology of HDLVEF, closely monitored β-blockade might be examined to attenuate the HDLVEF. Similarly, the trend toward association between CHF and HDLVEF is interesting and warrants further investigation.

## Conclusions

Compared with critically ill patients with NLVEF, the finding of HDLVEF in critically ill patients was associated with female sex, increased age, and diagnoses of hypertension and cancer. Patients with HDLVEF had increased 28-day mortality compared with those with normal EF in multivariate logistic regression analysis adjusting for age, sex, SOFA score, Elixhauser score of comorbidities, vasopressor use, and mechanical ventilation use. The presence of HDLVEF portended increased 28-day mortality and may be helpful as a gravity marker for prognosis of patients admitted to the ICU. Further research is warranted to better understand how these patients respond to common interventions in the ICU and to determine if pharmacologic modulation of HDLVEF improves outcomes.

## Key messages

- HDLVEF prevalence was 8.6 % in ICU patients who underwent TTE during their ICU admission.
- HDLVEF in the ICU has not been well described in the literature, especially on a large scale.
- The finding of HDLVEF was associated with female sex, increased age, hypertension, and cancer.
- Patients with HDLVEF were sicker and had increased adjusted 28-day mortality compared with the other patients.
- Further research is warranted to better understand how HDLVEF patients respond to common interventions in the ICU.

## Abbreviations

ACC: American College of Cardiology
CHF: congestive heart failure
CSRU: cardiac surgical resuscitation unit
DRG: Diagnosis-related group
EF: ejection fraction
HDLVEF: hyperdynamic left ventricular ejection fraction on echocardiogram
ICD-9: International Classification of Diseases–Ninth Revision
ICU: intensive care unit
IQR: interquartile range
IV: intravenous
LVEF: left ventricular ejection fraction
MAP: mean arterial pressure
MICU: medical intensive care unit
MIMIC II: Multiparameter Intelligent Monitoring in Intensive Care II, an ICU database
NLVEF: normal left ventricular ejection fraction on echocardiogram
SICU: surgical intensive care unit
SOFA: Sequential Organ Failure Assessment
TTE: transthoracic echocardiography
WBC: white blood cell count
